# Secondary hemophagocytic lymphohistiocytosis in a patient with rheumatoid arthritis and vasculitis: a case report and review of the literature

**DOI:** 10.31138/mjr.29.3.163

**Published:** 2018-09-27

**Authors:** Panagiotis Panagopoulos, Gkikas Katsifis

**Affiliations:** Rheumatology Clinic, Naval Hospital of Athens, Athens, Greece

**Keywords:** Hemophagocytic lymphohistiocytosis, rheumatoid vasculitis

## Abstract

Hemophagocytic lymphohistiocytosis (HLH) is a life-threatening disorder characterized by excessive systemic inflammation, caused by uncontrolled activation of lymphocytes and macrophages, which secrete increased amounts of cytokines. HLH may be caused by gene mutations (primary HLH) or associated with malignancy, immunodeficiency, infection or autoimmune disease (secondary HLH). A 58-year-old woman with seropositive rheumatoid arthritis (RA) presented with fever, ulcers on the left foot and in the intergluteal cleft and increased inflammation markers. Clinical and laboratory evaluation, combined with findings from intra-arterial digital subtraction angiography of the lower limbs, pointed towards the diagnosis of vasculitis. Intravenous administration of low-dose cyclophosphamide resulted in recession of fever and decrease of inflammation markers. However, the patient later developed pancytopenia, hepatomegaly, hyperferritinemia, hypofibrinogenemia and hypertriglyceridemia, while bone marrow aspiration demonstrated hemophagocytosis. The diagnosis of HLH was established. An extensive workup excluded malignancies, systemic infections and immunodeficiencies. HLH in our patient was attributed to activation of RA and presentation of vasculitis. Treatment with corticosteroids and intravenous immunoglobulin led to resolution of fever, correction of pancytopenia and complete healing of the ulcers. Timely diagnosis and treatment of HLH is highly important for a favorable outcome for the patients. Treatment of secondary HLH associated with autoimmune diseases involves corticosteroids and/or other immunomodulatory agents, such as intravenous immunoglobulin.

## INTRODUCTION

Hemophagocytic lymphohistiocytosis (HLH) is an immune-mediated disorder, which is characterised by uncontrolled systemic inflammation caused by unregulated proliferation and activation of lymphocytes and macrophages, secreting excessive amounts of cytokines. The pathologic hallmark of HLH is hemophagocytosis, a process by which histiocytes engulf blood cells and their precursors in bone marrow, spleen, lymph nodes or other tissues. Clinical presentation may include fever, cytopenia, hepatosplenomegaly, lymphadenopathy, skin rash, central nervous system dysfunction and may even lead to multi-organ failure.^1^ HLH may be primary or secondary. Primary HLH is caused by gene mutations affecting the function of natural killer (NK) and cytotoxic T cells and presents in infants and children. In secondary HLH, which may present at any age, there is usually a predisposing condition dysregulating immune system (such as malignancy, immunodeficiency, chronic infection or autoimmune disease) and/or a trigger (such as an infection or a flare of autoimmune disease). When it is associated with autoimmune diseases, HLH is also called macrophage activation syndrome (MAS).^2^

HLH is a rare disease, although its diagnosis has increased lately due to increased awareness. Indeed, a retrospective study from Sweden in the early 1990s reported an incidence of primary HLH of 1.2 per 1,000,000 children per year,^3^ whereas a more recent study from Wisconsin in early 2010s reported a much greater incidence of 1.5 children per 100,000 per year.^4^ A nationwide survey in Japan assessing both primary and secondary HLH estimated an incidence of 1 in 800,000 per year,^5^ while a single-center population-based retrospective analysis from Sweden estimated an annual incidence of malignancy-associated HLH in adulthood of 0.36 per 100,000 individuals.^6^

The prognosis of HLH is poor. A review of 1109 cases of secondary HLH estimated the mortality rate in adults at 41%. Prognosis of secondary HLH strongly depends on the underlying causes: patients with malignancy have the poorest outcome, while patients with primary viral infection or autoimmune disease show better results.^7^

We present a case of secondary HLH in a patient with rheumatoid arthritis (RA) who presented with fever and vasculitic ulcers on the left foot and in the intergluteal cleft and later developed pancytopenia, hepatomegaly and hemophagocytosis in bone marrow aspirate. We also give a brief review of the literature on diagnosis and treatment of HLH.

## CASE REPORT

A 58-year-old woman of Polish origin with rheumatoid arthritis presented with fever (temperatures to 38.5°C measured at home) and ulcers on the dorsum of the left foot and in the intergluteal cleft. She had seropositive rheumatoid arthritis for 15 years, which had been difficult to treat and for which she had received treatment with various biological agents: adalimumab, certolizumab pegol and tocilizumab. At the time of presentation, the RA treatment was methylprednisolone 2mg/day, methotrexate 15mg/week and rituximab 2g/6 months. She also had a past medical history of deep venous thrombosis in both legs, hypothyroidism and osteoporosis. Clinical examination on presentation was unremarkable with the exception of the infected ulcers on the dorsum of the left foot and in the intergluteal cleft. Laboratory investigations were remarkable for elevated erythrocyte sedimentation rate (ESR: 34mm) and C-reactive protein (CRP: 83mg/l), while blood cultures were negative. For the treatment of the infected ulcers, ciprofloxacin and clindamycin were administered intravenously (iv) for 10 days and surgical debridement was performed frequently. Eleven days after admission, the patient was discharged on ciprofloxacin and clindamycin per os and hyperbaric oxygen therapy was scheduled in order to facilitate healing of the ulcers.

**Figure 1.**
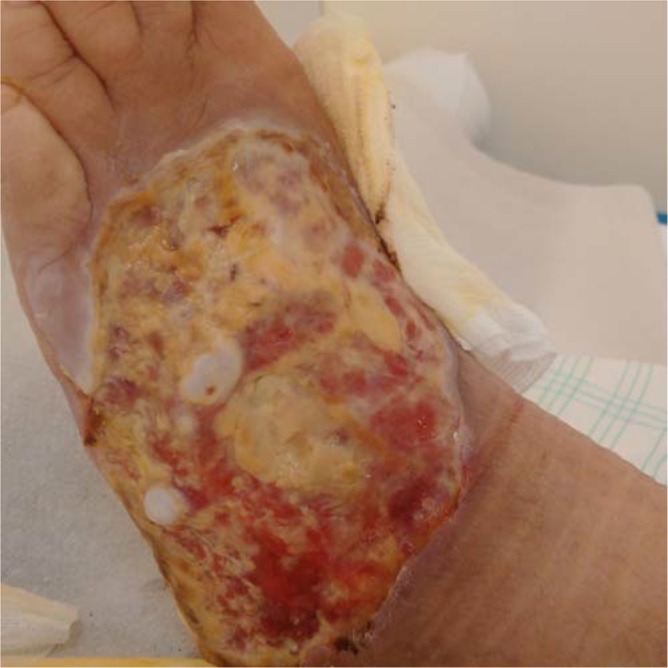
Ulcer on the dorsum of the patient’s left foot.

**Figure 2.**
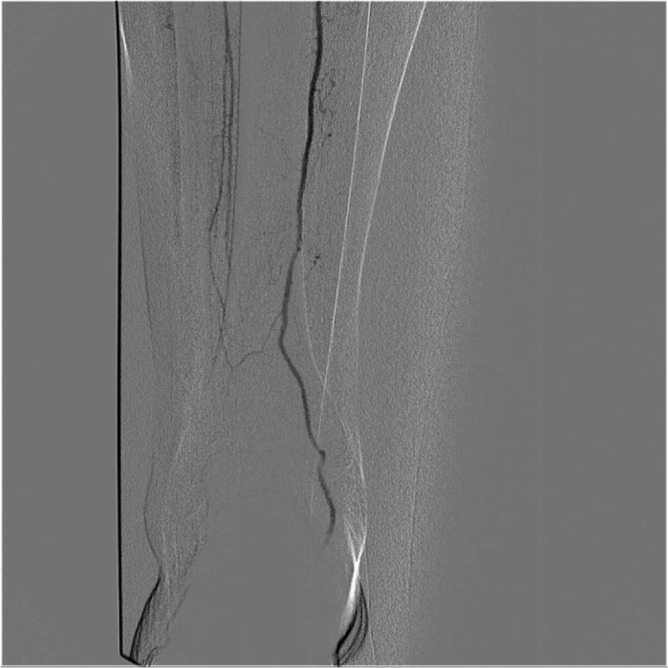
Intra-arterial digital subtraction angiography of left lower limb showing significant luminal narrowing of left peroneal artery.

However, 24 days after discharge the fever relapsed while the ulcers were not improving (**Figure 1**), so the patient was admitted again. Apart from fever up to 38.3°C, clinical examination this time revealed subcutaneous nodules on both forearms, while inflammation markers had increased (ESR: 44mm, CRP: 113mg/l). Tissue from the ulcer of the foot was received for culture, which was positive for E. coli. Blood cultures were once again negative. Magnetic resonance imaging (MRI) of left foot was performed in order to exclude the possibility of osteomyelitis and revealed inflammation of soft tissues. Concerning the etiology of ulcers, vasculitis was considered the most likely diagnosis. To delineate the underlying vascular pathology intra-arterial digital subtraction angiography (i.a. DSA) of the lower limbs was performed. DSA demonstrated luminal narrowing of peroneal (fibular) arteries bilaterally, which was significant in left peroneal artery (**Figure 2**). These findings supported the diagnosis of vasculitis. Additional immunological workup demonstrated positive rheumatoid factor (RF) and antinuclear antibodies (ANA) and negative anti-double stranded deoxyribonucleic acid (anti-dsDNA) antibodies, antibodies to extractable nuclear antigens (anti-ENA), perinuclear and cytoplasmic anti-neutrophil cytoplasmic antibodies (p-ANCA, c-ANCA) and cryoglobulins. Therefore, low-dose cyclophosphamide was administered iv (125mg and 250mg, a week apart), resulting in recession of fever and nodules and a decrease of ESR and CRP. In addition, meropenem and clindamycin were administered iv, following the antibiogram of soft tissue culture from the ulcer, and frequent surgical debridement was performed. Furthermore, the patient underwent hyperbaric oxygen therapy daily. Treatment resulted in improvement of the ulcers, thus the patient was discharged 28 days after second admission and a re-evaluation visit was scheduled.

**Figure 3.**
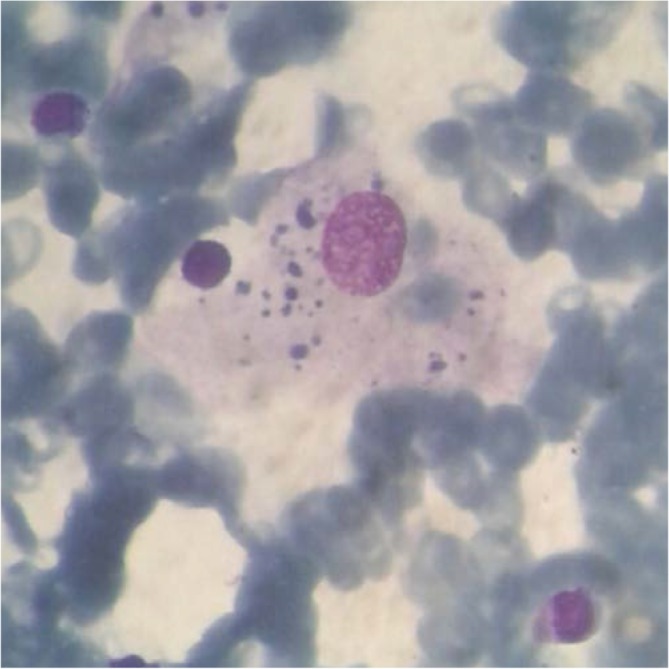
Bone marrow aspirate showing a macrophage with hemophagocytosis.

Seven days after second discharge the patient was readmitted, because of fever relapse, agranulocytosis (absolute neutrophil count: 60 cells per microlitre) and oral candidiasis. Inflammation markers had increased even more (ESR: 117mm/h, CRP: 418mg/l), while blood cultures were once again negative. Chest and abdominal CT scan was performed for the exclusion of malignancy and lymphoproliferative disorders and demonstrated only mild hepatomegaly. Hemoglobin and platelet count gradually decreased (**Table 1**) and the patient developed pancytopenia on the 4th day of admission. Additional laboratory data showed hyperferritinemia (>1000 mg/dL), hypofibrinogenemia and hypertriglyceridemia. Bone marrow aspiration was performed and demonstrated hemophagocytosis, agranulocytosis and megakaryocytic hypoplasia (**Figure 3**). Repeated blood cultures and an extensive workout for infection including serology for Epstein-Barr virus (EBV), cytomegalovirus (CMV), herpes simplex virus (HSV), hepatitis B virus (HBV), hepatitis C virus (HCV), human immunodeficiency virus (HIV), toxoplasma and brucella were negative.

**Table 1:** Laboratory data of patient’s third admission.

**Characteristic (reference laboratory value**)	**Admission**	**Day 4**	**Day 5 (steroid+IVIG initiation)**	**Day 7**	**Day 11**	**Day 22**	**Day 29 (discharge)**	**1 month post-discharge**
Hemoglobin (12–16 g/dl)	10.1	8.2	8.1	7.3	7.8	8.7	10.0	12.2
White blood cell (4000–10000/μl)	660	840	730	990	1230	10640	6860	6640
Neutrophil (1600–2500/μl)	60	40	50	100	200	5500	3600	3800
Lymphocyte (1500–3600/μl)	580	750	630	840	950	3770	2400	2220
Platelet (150–450 K/ml)	171	50	15	4	35	42	111	160
ESR (0–20 mm)	117	110			100	20	35	5
CRP (0–7 mg/l)	418	338	256	85	28	2.4	1	3.95
Creatinine (0.6–1.1 mg/dl)	0.75	0.71	0.65	0.52	0.64	0.8	0.93	0.77
Aspartate aminotransferase (5–37 U/l)	27.7	9.8	13.3	11.4	10.0	16.0	13.0	16.0
Alanine aminotransferase (5–35 U/l)	21.5	12.5	10.9	10.8	10.9	18.0	20.0	29.0
Triglycerides (35–150 mg/dl)	137			317				182
Ferritin (10–120 ng/ml)			1072					83
Immunoglobulin G (700–1600 mg/gl)	693							874
International Normalized Ratio	3.6				1.14		0.99	2.15
Fibrinogen (200–450 mg/dl)					144		141	292
Urinalysis	normal				normal	normal		

The presentation of fever, pancytopenia and hemophagocytosis in bone marrow, following a disease flare in the form of vasculitis, in combination with hepatomegaly, hyperferritinemia, hypofibrinogenemia and hypertriglyceridemia, in the setting of a negative infectious and malignant disease workup, was compatible with the diagnosis of secondary hemophagocytic lymphohistiocytosis (HLH). Indeed, the patient fulfilled multiple Histiocyte Society HLH-2004 diagnostic criteria (**Table 2**), including fever, cytopenias affecting all 3 lineages, hypertriglyceridemia, hypofibrinogenemia, hemophagocytosis in bone marrow and hyperferritinemia.

**Table 2:** The HLH-2004 diagnostic criteria.

The diagnosis of HLH may be established by a molecular diagnosis consistent with HLH or five out of the eight following criteria:
1. Fever
2. Splenomegaly
3. Cytopenias (affecting ≥2 of 3 lineages in the peripheral blood):
a. Hemoglobin <9 g/dl
b. Platelets <100 × 10^9^/l
c. Neutrophils <1.0 × 10^9^/l
4. Hypertriglyceridemia and/or hypofibrinogenemia:
a. Fasting triglycerides ≥265 mg/dl
b. Fibrinogen ≤1.5 g/L
5. Hemophagocytosis in bone marrow or spleen or lymph nodes
6. Low or absent NK-cell activity
7. Ferritin ≥500 ng/mL
8. Soluble CD25 (i.e., soluble IL-2 receptor) ≥2,400 U/ml

For the treatment of secondary HLH, corticosteroids and intravenous immunoglobulin (IVIG) were initiated. Methylprednisolone was administered at 125mg/day intravenously for 3 days, followed by 40mg/day orally. IVIG was administered at 500mg/kg daily for 10 days. The patient was also started on filgrastim subcutaneously, a granulocyte colony-stimulating factor (G-CSF) analog. In addition, empiric broad-spectrum antibiotic therapy for neutropenic fever was initiated, including ceftazidime, clindamycin, vancomycin and caspofungin. Trimethoprim/sulfamethoxazole was also administered for pneumocystis prophylaxis, as well as acyclovir for prevention of reactivation of herpes viruses. Supportive treatment included red blood cell and platelet transfusions and parenteral nutrition.

**Figure 4.**
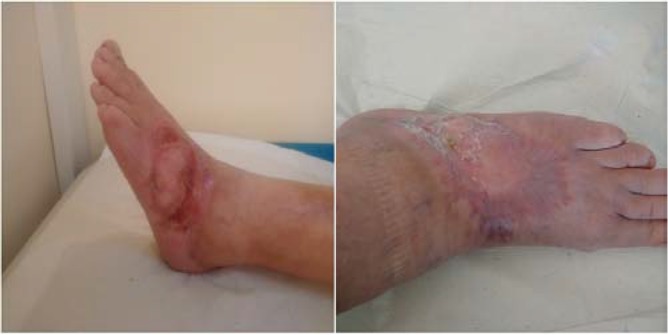
Completely healed ulcer on the dorsum of left foot one month after patient’s third discharge.

The treatment led to fever resolution and gradual improvement of anemia, neutropenia, thrombocytopenia and rest laboratory data (**Table 1**). One month after third discharge, the ulcers on the dorsum of left foot and in the intergluteal cleft had healed completely (**Figure 4**) and complete blood count had returned to normal. The patient is currently on treatment with rituximab 2g/6 months for RA.

## DISCUSSION

Diagnosis of hemophagocytic lymphohistiocytosis (HLH) is established by a combination of symptoms, signs and laboratory abnormalities. The Histiocyte Society has proposed diagnostic criteria for HLH, which were used for the pediatric HLH-2004 study (**Table 2**).^8^ These criteria have been developed for the pediatric population, but they have been widely used in adult patients with HLH. Their limitations include limited accessibility of molecular testing, NK function and soluble CD25, as well as different presenting features of HLH in adults. Recently, Fardet et al. proposed a new set of criteria for diagnosis of secondary HLH. They developed the H-Score which assigns points to 9 variables and predicts the probability of having HLH (**Table 3**). Fardet et al. proposed that the best cutoff value for the diagnosis of HLH is 169, which corresponds to a sensitivity of 93% and a specificity of 86%.^9^

**Table 3:** The H-Score.

**Variable**	**Points**
Known underlying immunosuppression	0 (no) or 18 (yes)
Temperature (°C)	0 (<38.4), 33 (38.4–39.4), or 49 (>39.4)
Organomegaly	0 (no), 23 (hepatomegaly or splenomegaly), or 38 (hepatomegaly and splenomegaly)
Number of cytopenias	0 (1 lineage), 24 (2 lineages), or 34 (3 lineages)
Ferritin (ng/ml)	0 (<2,000), 35 (2,000–6,000), or 50 (>6,000)
Triglyceride (mmoles/l)	0 (<1.5), 44 (1.5–4), or 64 (>4)
Fibrinogen (g/l)	0 (>2.5) or 30 (≤2.5)
Serum aspartate aminotransferase (U/l)	0 (<30) or 19 (≥30)
Hemophagocytosis features on bone marrow aspirate	0 (no) or 35 (yes)

Hemophagocytosis may be the pathologic hallmark of HLH, however it lacks both sensitivity and specificity.^10^ Moreover, the amount of hemophagocytosis in bone marrow aspirate does not always correlate with the probability of HLH.^11^ Thus, diagnosis of HLH cannot be based solely on histological findings of hemophagocytosis. It requires good clinical judgement and an overall assessment of the patient.^12^

Besides, although most patients with HLH present with hyperferritinemia, this finding is not specific for HLH. The HLH-2004 criteria include ferritin ≥500 ng/mL, based on the HLH-94 study which demonstrated a sensitivity of 84% in pediatric population for this cutoff value.^8^ A more recent study in pediatric patients showed that a higher ferritin threshold of ≥10,000 ng/mL was 90% sensitive and 96% specific for diagnosis of HLH.^13^ On the other hand, recent studies demonstrated that marked hyperferritinemia is not specific for HLH in adult population.^14,15^

Our patient fulfilled 5 of 8 HLH-2004 criteria and also had a diagnostic H-Score. Previous immunosuppressive therapy for RA, maximal temperature of 38.5ºC, hepatomegaly, cytopenia affecting all 3 lineages, a minimal fibrinogen of 141 mg/dl and hemophagocytosis on bone marrow aspirate correspond to an H-Score of 217 and a diagnostic probability >93%.

Secondary HLH is usually attributed to conditions undermining immune system. Viral infections (such as EBV, CMV, HSV, HIV, HBV or HCV) are common causative factors. Other conditions predisposing to HLH include malignancies (mainly hematological neoplasms, especially lymphoma) and autoimmune diseases (such as systemic lupus erythematosus, adult-onset Still’s disease and rheumatoid arthritis).^7^ In our patient, infections, malignancies and immunodeficiencies were excluded by an extensive workup, including repeated blood cultures, serology testing for multiple viruses, chest and abdominal CT, complete blood count, bone marrow aspiration and immunoglobulin measurement. Thus, RA activation with vasculitis seemed to be the cause for triggering HLH in our patient. In such cases, when HLH is attributed to autoimmune diseases, is called MAS (macrophage activation syndrome).

The goal of the treatment of HLH is to control the overactive immune system and suppress excessive inflammation. The Histiocyte Society has proposed the etoposide-based HLH-2004 protocol for the treatment of primary HLH, which includes an 8-week initial therapy with dexamethasone, etoposide and cyclosporine, as well as intrathecal administration of methotrexate and hydrocortisone in case of central nervous system involvement. Patients who fail to respond are candidates for hematopoietic cell transplantation.^8^ In secondary HLH, it is important to first identify and treat the primary cause (infection, malignancy or autoimmune disease). A number of treatments have been used in secondary HLH; including corticosteroids, cyclosporine, methotrexate, cyclophosphamide, intravenous immunoglobulin (IVIG), the HLH-2004 protocol and biological agents (anakinra, rituximab, infliximab, etanercept).^7,16^ There are no randomized, controlled clinical trials, thus treatment decisions depend on clinical experience and expert opinion.^7^ Moreover, the efficacy of HLH-2004 protocol has not been proven in adult patients and secondary HLH. Although a retrospective study of 162 adults with secondary HLH found that treatment strategies containing etoposide improved survival of patients,^17^ another retrospective study of 103 cases of secondary HLH in adults showed that treatment with etoposide did not affect prognosis.^18^ Besides, in patients with HLH secondary to autoimmune diseases (MAS), treatment of the underlying autoimmune disorder with corticosteroids and/or other agents, such as IVIG, is usually effective. Indeed, in a retrospective study with 30 patients with an underlying autoimmune disease and HLH/MAS, initial monotherapy with corticosteroids was sufficient for nearly half the patients. Addition of cyclosporine, cyclophosphamide or tacrolimus led to remission 80% of the patients.^19^ Moreover, retrospective study with 38 patients with secondary HLH showed that treatment with corticosteroids and/or IVIG is sufficient for patients with HLH/MAS with an underlying autoimmune disease.^20^

Concerning our patient, RA was the underlying disorder and activation of the disease with the presence of vasculitis was the trigger of HLH. Therefore, we opted for a treatment with corticosteroids and IVIG, which proved to be effective, since it led to immediate resolution of fever, gradual correction of cytopenias and complete healing of the vasculitic ulcers.

## ABBREVIATIONS

CRP: C-reactive Protein
ESR: Erythrocyte Sedimentation Rate
HLH: Hemophagocytic Lymphohistiocytosis
MAS: Macrophage Activation Syndrome
RA: Rheumatoid Arthritis
